# Unravelling the drastic range retraction of an emblematic songbird of North Africa: potential threats to Afro-Palearctic migratory birds

**DOI:** 10.1038/s41598-017-01103-w

**Published:** 2017-04-24

**Authors:** Rassim Khelifa, Rabah Zebsa, Hichem Amari, Mohammed Khalil Mellal, Soufyane Bensouilah, Abdeldjalil Laouar, Hayat Mahdjoub

**Affiliations:** 1 Department of Evolutionary Biology and Environmental Studies, University of Zurich, Winterthurerstrasse 190, CH-8057 Zurich, Switzerland; 2 Department of Nature and Life Sciences, Faculty of Nature and Life Sciences and Earth and Universe Sciences, University of 08 May 1945, Guelma, 24000 Algeria; 3 Laboratory of Functional and Evolutionary Ecology, Department of Biology, University of Chadli Bendjedid, El Tarf, 36000 Algeria; 4 Laboratory of Marine and Coastal Environments Ecobiology, Department of Biology, Badji Mokhtar University, Annaba, BP 12 23000 Algeria; 5 Laboratoire de Zoology, Université de Mons, 20, place du Parc, B7000 Mons, Belgium

## Abstract

Understanding how culture may influence biodiversity is fundamental to ensure effective conservation, especially when the practice is local but the implications are global. Despite that, little effort has been devoted to documenting cases of culturally-related biodiversity loss. Here, we investigate the cultural domestication of the European goldfinch (*Carduelis carduelis*) in western Maghreb (Morocco, Algeria and Tunisia) and the effects of long-term poaching of wild populations (1990–2016) on range distribution, socio-economic value, international trading and potential collateral damage on Afro-Palearctic migratory birds. On average, we found that the European goldfinch lost 56.7% of its distribution range in the region which led to the increase of its economic value and establishment of international trading network in western Maghreb. One goldfinch is currently worth nearly a third of the average monthly income in the region. There has been a major change in poaching method around 2010, where poachers started to use mist nets to capture the species. Nearly a third of the 16 bird species captured as by-catch of the European goldfinch poaching are migratory, of which one became regularly sold as cage-bird. These results suggest that Afro-Palearctic migratory birds could be under serious by-catch threat.

## Introduction

Species overexploitation for wildlife trade is a major global threat to biodiversity, particularly birds^1, 2^. Many species of birds are targets of poaching due to their high economic and cultural value^3^, threatening wild populations and ecosystem functioning^4, 5^. Nearly half of the extant and extinct bird species have been utilized by humans for various purposes, especially for domestication as pets^1^. These species are often not exploited sustainably^6^, and many are threatened by extinction^7^. Although poaching is sometimes species-specific and tends to have local consequences on target species^8–10^, it can also be non-selective with substantial consequences on a large number of local and migratory species^11^.

The Mediterranean has recently been identified as a danger zone for migratory birds where 11–36 million individuals per year may be killed or taken illegally, and one of the most important reasons is for use as cage-birds^11^. This is an alarming issue for global avian diversity since one of the world's major flyways, the Palearctic-African flyway^12^, crosses the Mediterranean where an estimated number of 2.1 billion Afro-Palearctic birds migrate every year back and forth from breeding to wintering grounds^13^. Moreover, migratory birds do not only fly through the Mediterranean region but also spend several days in stop-overs to rest and eat^14, 15^ which increases their vulnerability to poaching. In North Africa, and particularly western Maghreb (Morocco, Algeria, and Tunisia), stop-over areas are known to be suitable for fat accumulation because of the availability of food and water^14^, and thus they potentially host a large number of migratory birds every year. However, the cultural practice of trapping the European goldfinch (*Carduelis carduelis*) for domestication in this region is widespread^16^. Yet, the potential effects of the local poaching practice on Afro-Palearctic migratory birds has not been investigated.

The European goldfinch is a widespread songbird, considered as one of the most abundant passerine species in the Palearctic^17^. The species has been domesticated in cages for a long time in the Palearctic where it is very popular and holds important cultural significance^3, 18–20^. It has been recognized that more than 400 paintings, particularly those of Carel Fabritius included the goldfinch as symbol of spirit, resurrection, sacrifice or death^21^. In North Africa, the use of a goldfinch as a pet goes back to the Umayyad dynasty around 700 AD and reached the region through the expansion of the Umayyad Caliphate. Nowadays, it is part of the culture of this region to buy and raise goldfinches in cages for their elegant colors and large repertoire of singing. In western Maghreb (Morocco, Algeria, and Tunisia), the industrial poaching of the species started in the early 1990s. Following a deep economic crisis in these countries, goldfinch poaching became a job and a hobby for local people who trapped the species all year long, even during the reproductive season. Bird trapping and killing has been prohibited in western Maghreb since 2004 in Algeria^22^, 2007 in Tunisia^23^, and 1962 in Morocco^24^, but the application of conservation laws is often not respected and their effectiveness is thus questionable^11^. Here, we document the effects of the long-term poaching on local populations of European goldfinch to reveal the consequences of overexploitation of natural populations on abundant animal species.

Goldfinch poaching has been conducted with various methods including lime sticks, cage traps and ground nets. However, from around 2010, when the species became particularly rare, poachers started to use mist nets to increase the chances of capture. Mist nets are known to intercept all birds that fly through them^25^. Even when the birds are released, this method may induce injuries and lower the survival probability of individuals^26^. In addition, this poaching method is potentially threatening to many migratory birds. Indeed, western Maghreb lies in a major migration flyway where millions of birds fly through this region or make stopovers during their migration^13, 27^. Therefore, the understanding of the potential damages caused by the European goldfinch's poaching in western Maghreb may shed light on why many Afro-Palearctic migrant birds have declined during the past two decades^28^.

The causes of changes in the geographic distribution of the European goldfinch in western Maghreb and the potential consequences of its intensive poaching on Afro-Palearctic migratory species have not been surveyed yet. Here, we first combined observations from our field visits with questionnaire data of (ex-)poachers to build historical presence/absence data on the species in western Maghreb and assess the changes of its distribution with a dynamic occupancy model^29, 30^. Second, we estimate the current population size of European goldfinch in captivity by conducting a survey that aims at estimating the number of individuals held in cage per family to reveal the cultural importance of the species and the intensity of poaching. Third, we evaluate the international trading network of the species in western Maghreb using seizure data from Algerian conservation authorities and gendarmerie. Finally, we highlight the potential collateral damage of European goldfinch poaching on local and Afro-Palearctic migratory birds based on information gathered from bird markets and ex-poachers.

## Results

### Dynamics of occurrence

Of the 237 selected grids (25 × 25 km²) which cover the potential habitat of the European goldfinch in western Maghreb, the species was observed in 115 grids (48.5%, n = 237) during 1990–1991 and in only 37 grids (15.6%, n = 237) during 2015–2016. The highest decline was recorded in Tunisia and Algeria where the number of occupied grids decreased from 16 (38.1%, n = 42) in 1990–1991 to 0 (0%, n = 42) in 2015–2016 and from 58 (48.7%, n = 119) in 1990–1991 to 3 (2.5%, n = 119) in 2015–2016, respectively. In Morocco, the number of occupied grids declined from 41 (53.9%, n = 76) in 1990–1991 to 34 (44.7%, n = 76) in 2015–2016.

To estimate the changes in range distribution, we used dynamic occupancy models which account for detection probability. The dynamic occupancy model showed strong evidence for time-dependence in detection probability. Detection probability was influenced by vegetation cover (linear and quadratic), elevation (linear and quadratic), and by the interaction terms between elevation and vegetation cover (vegetation-by-elevation and vegetation-by-elevation²). Initial occupancy was affected by elevation (linear and quadratic) and vegetation cover. Extinction probability was best explained by longitude (linear and quadratic) and elevation, whereas colonization probability was constant (Table 1; Supplementary Table 1).

**Table 1:** Summary results of the best dynamic occupancy model for the range dynamics of the European goldfinch (*Carduelis carduelis*) in western Maghreb from 1990–1991 to 2015–2016.

	Estimate	SE	z	P(>|z|)
**Initial occupancy probability**				
Intercept	−0.855	0.550	−1.56	0.1200
vegetation	8.949	1.952	4.58	<0.0001
elev	4.962	1.168	4.25	<0.0001
elev²	−2.612	0.722	−3.62	0.0003
**Colonization probability**				
Intercept	−10.7	51.2	−0.209	0.835
**Extinction probability**				
Intercept	−3.39	1.76	−1.93	0.0535
long	17.89	7.90	2.26	0.0236
long²	17.29	7.64	2.26	0.0237
elev	2.96	1.68	1.76	0.0784
**Detection probability**				
Intercept	3.85	1.194	3.23	0.00125
Period (2015–2016)	−4.08	1.042	−3.92	<0.0001
vegetation	5.40	2.197	2.46	0.01400
vegetation²	−3.08	1.151	−2.68	0.00747
elev	4.09	1.443	2.83	0.00462
elev²	−2.70	1.112	−2.43	0.01510
vegetation:elev	−3.43	1.059	−3.24	0.00120
vegetation:elev²	1.57	0.746	2.11	0.03480

Covariates include vegetation cover (vegetation), elevation (elev), longitude (long), and period (primary sampling period). Colons indicates interactions.

**Figure 1:**
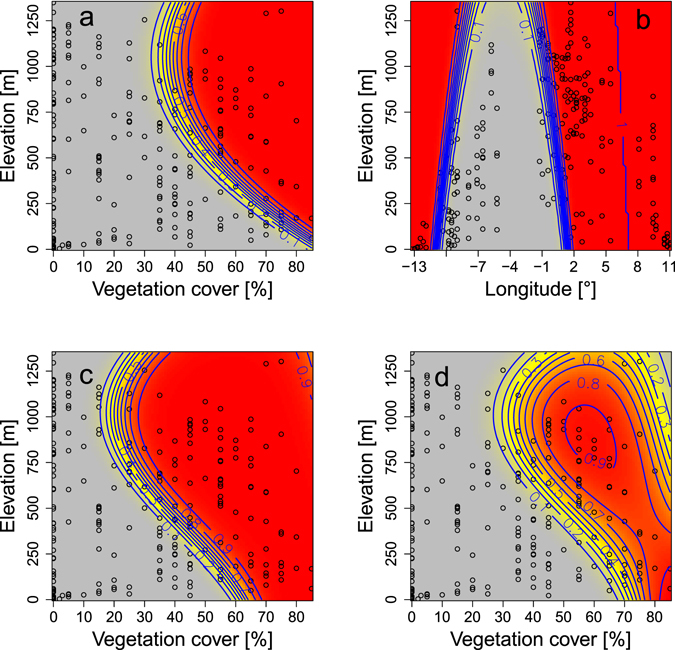
Predicted relationship of three parameters of the dynamic occupancy model with covariates (vegetation cover, elevation and longitude) for the range dynamics of the European goldfinch (*Carduelis carduelis*) in western Maghreb from 1990–1991 to 2015–2016. (**a**) Predicted response of occupancy probability in 1990–1991. (**b**) Extinction probability. (**c**) Detection probability in 1990–1991. (**d**) Detection probability in 2015–2016. The gradient of colour from grey to red corresponds to a range of low to high probability. Values of observed covariates at 237 sample plots are presented as circles.

Initial occupancy (year 1990–1991) was greatest at high vegetation cover in mid-high elevations (Fig. 1a). Extinction probability was lowest at a range of longitude situated in the west (Morocco) but this range shrank slightly with elevation (Fig. 1b). Detection probability was overall higher in the first sampling period (1990–1991) than in the second (2015–2016). In 1990–1991, detection probability was highest at low elevation with high vegetation cover and at mid-high elevation with relatively low to high vegetation cover (Fig. 1c). In 2015–2016, high detection probabilities were restricted to low elevations with very high vegetation cover and high elevations with intermediate to high vegetation cover (Fig. 1d).

**Figure 2:**
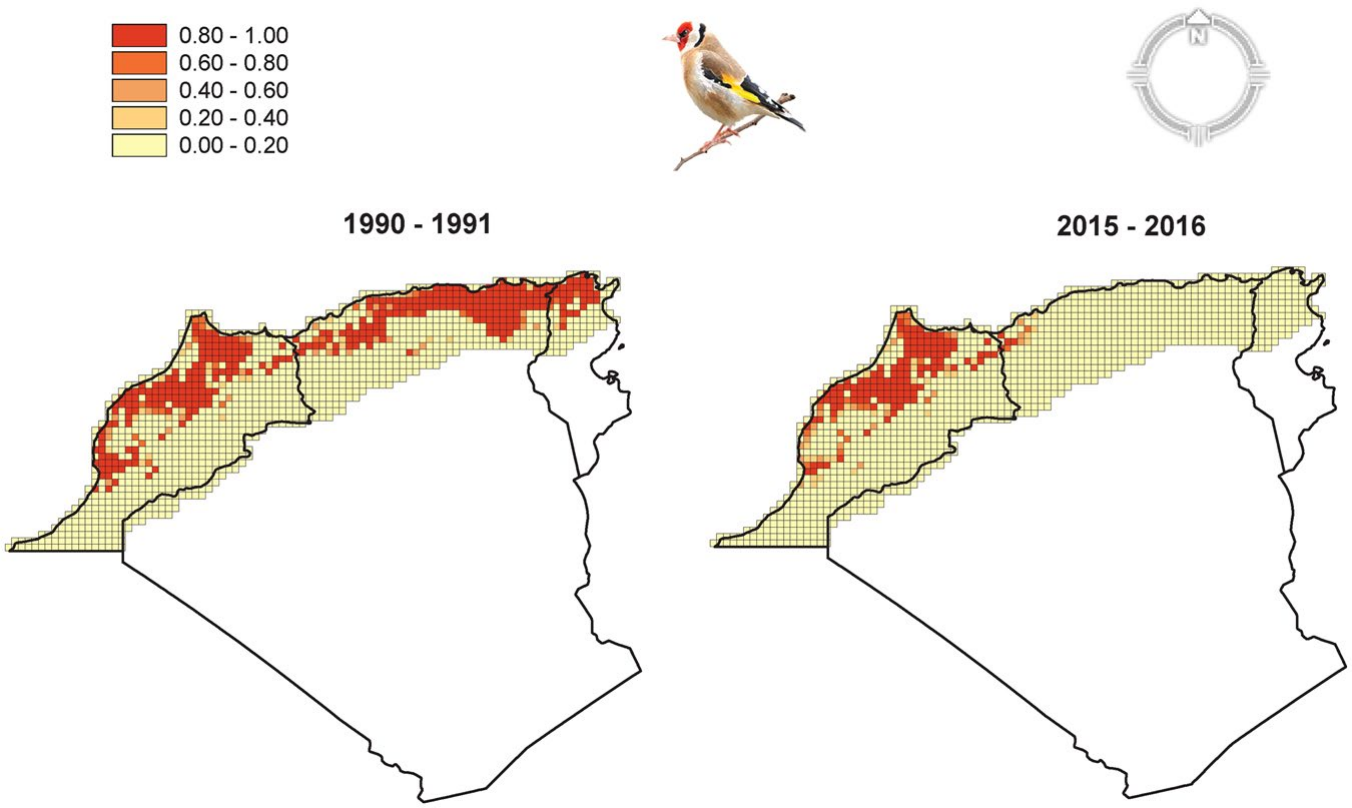
Dynamics of occupancy probability before (1990–1991) and after (2015–2016) the industrial poaching of the wild European goldfinch in western Maghreb. Occupancy probabilities were calculated based on the best dynamic occupancy model (see results). The gradient of color from yellow to red corresponds to a range of low to high occupancy probability. One grid is 25 × 25 km². The grids do not cover the entire geographic area of the three countries because areas where the species is unlikely to exist (desert) were excluded. The maps were created with MapInfo Professional software (version 15.0, http://www.pitneybowes.com/us/location-intelligence/geographic-information-systems/mapinfo-pro.html).

Based on our best model, we predicted the occurrence of the species on 1325 grids covering the western Maghreb region, excluding Sahara. We summed the predicted values of occupancy over the study area and found an estimated distribution area prior to industrial poaching (1990–1991) of 236725 km² (95% CI: 221834–264911 km²). In 2015–2016, the distribution range retracted to 102548 km² (95% CI: 126300.7–142635 km²), which corresponds to a loss of 56.7% of the home range in western Maghreb (Fig. 2).

### Temporal pattern of prices

**Figure 3:**
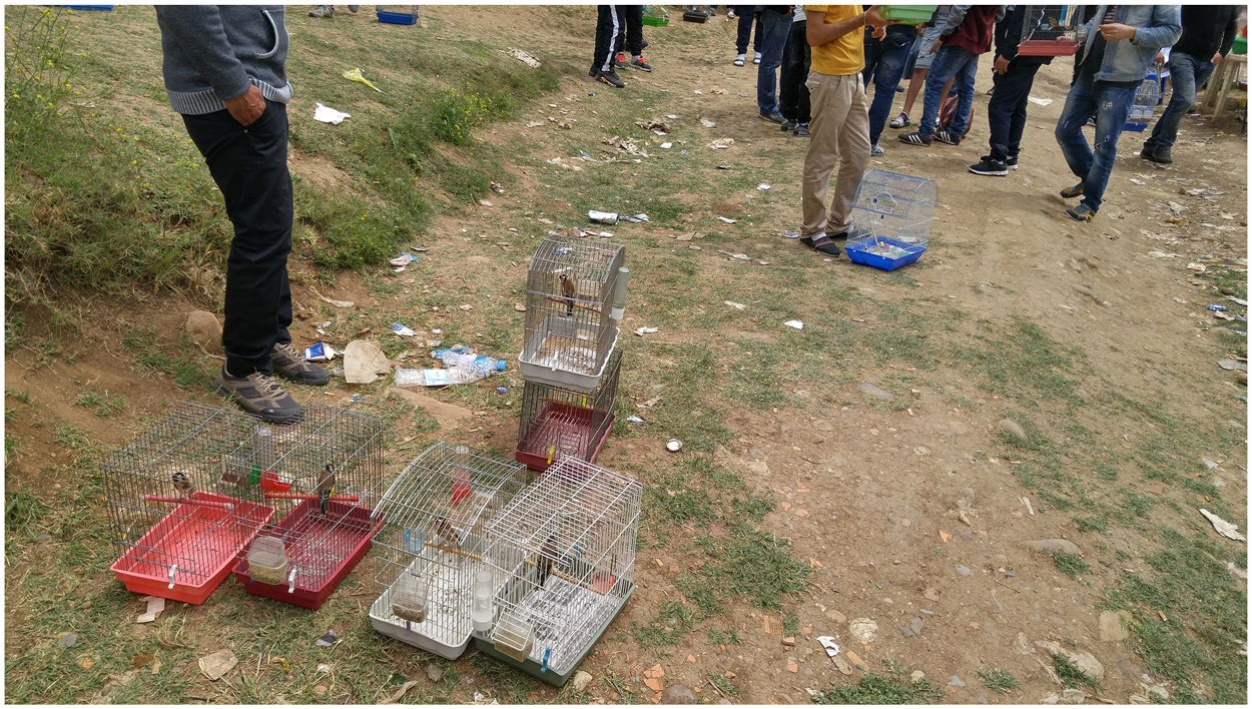
Illegal trading of the European goldfinch in the flea market of Algeria (Guelma 2016).

**Figure 4:**
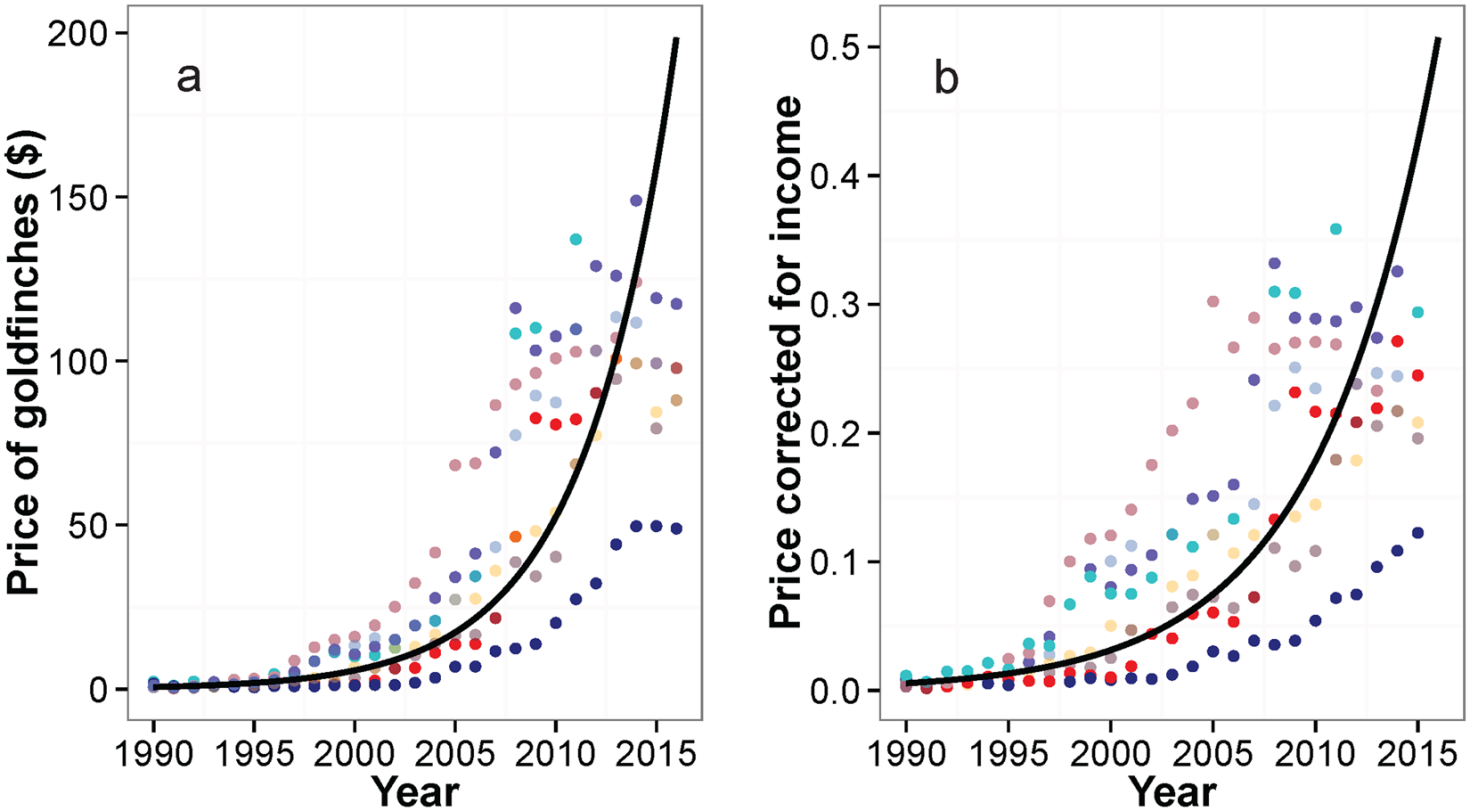
Temporal pattern of the price of the European goldfinch (*Carduelis carduelis*) in Algeria. (**a**) Price of adult male. (**b**) Price of adult male corrected for the monthly per capita income. The exponential curve in (**a**) and (**b**) represents the average across provinces. Prices were converted to US dollars ($). Colors refer to provinces; Pink: Algiers, Cyan: Annaba, Blue: Blida, Red: Guelma, Purple: Jijel, Grey: Oran, Yellow: Skikda, and Beige: Souk Ahras.

Our analysis of the temporal pattern of the price of the European goldfinch was based on information collected from the market of eight Algerian provinces between 1990 and 2016 (Fig. 3). The temporal pattern of the price of the species showed an exponential growth (R² = 0.94, P < 0.0001; Fig. 4a), revealing a very rapid increase during 2005–2016. On average (across provinces), the price increased 92.2 times from $1.08 ± 0.70 in 1990–1994 to $99.9 in 2011–2016. By correcting the price by the monthly per capita income (PCI), it still showed an exponential growth over time (R² = 0.89, P < 0.0001; Fig. 4b). When averaged across provinces, PCI increased 32.26 times from 0.007 ± 0.005 in 1990–1994 to 0.23 ± 0.06 in 2011–2016.

### Domestic population estimation

We investigated the number of goldfinches owned by 2721 families distributed in 15 Algerian provinces in 2016. A total of 2517 goldfinches was counted, which corresponds to an average of 0.925 (95% CI: 0.841–1.008) goldfinch per family (Supplementary Fig. 1). The total number of European goldfinch in captivity in Algeria was estimated by multiplying the total number of families (see Supplementary Material) by the average number of European goldfinches in captivity from the sample. We obtained an estimated total of 6.35 million (95% CI: 5.78–6.92 million) captive European goldfinches in Algeria, and 15.6 million (95% CI: 14.2–17.0 million) in the western Maghreb. Surprisingly, we found that only a proportion of 0.03 (95% CI: 0.029–0.043) of owners carry out in-cage breeding which suggests that traders rely on wild populations of the species to meet people’s needs. However, the quasi-extinct wild populations of European goldfinch in Algeria and Tunisia are far from being enough to supply people with wild European goldfinches, and thus most domestic individuals most likely come from illegal importation.

### International trading

**Figure 5:**
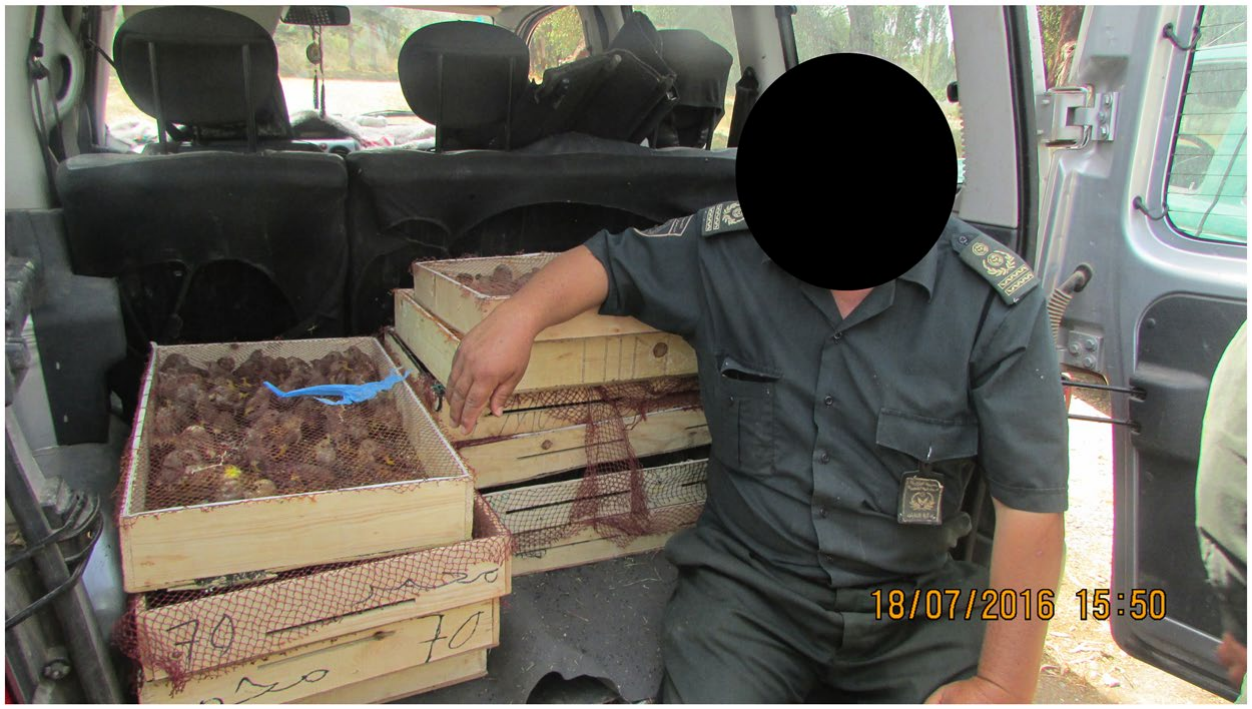
Apprehension of a thousand European goldfinches (*Carduelis carduelis*) by conservation authorities for illegal trading in Northwest Algeria near the Moroccan border (Oran, 2016).

**Figure 6:**
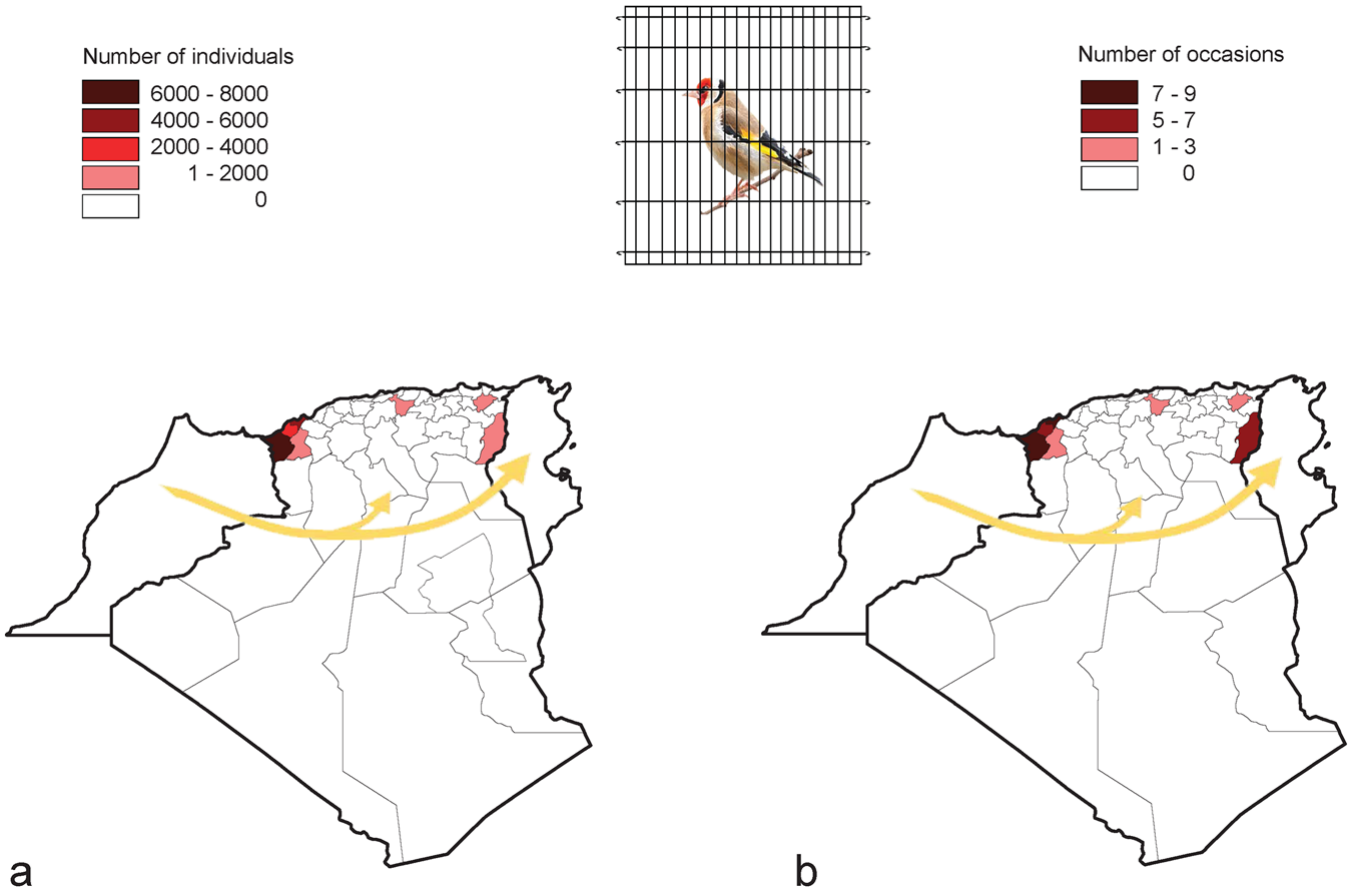
Number of individuals (**a**) and occasions (**b**) of interceptions of the European goldfinch (*Carduelis carduelis*) by authorities in Algeria. The yellow arrow indicates the direction of European goldfinch exportation for international trading. Algeria is divided into administrative provinces. The map was made with MapInfo Professional software (version 15.0, http://www.pitneybowes.com/us/location-intelligence/geographic-information-systems/mapinfo-pro.html).

Between June 2008 and July 2016, a total of 18569 European goldfinches were intercepted on 30 occasions by the Algerian authorities (Fig. 5). All these birds originated from Morocco and illegally crossed the Algerian border. These goldfinches were intercepted in seven Algerian provinces located in northwestern Algeria near the Moroccan border (4 provinces), in the north (1 province), and northeastern Algeria near the Tunisian border (2 provinces). Of the total number of European goldfinches, 98.1% were intended to be traded in Algeria and 1.9% were heading to Tunisia (Fig. 6a,b). The mean batch size of transported European goldfinches was 618.9 ± 521.3 goldfinches (n = 30; range = 65–2020 birds).

### Bycatch

**Figure 7:**
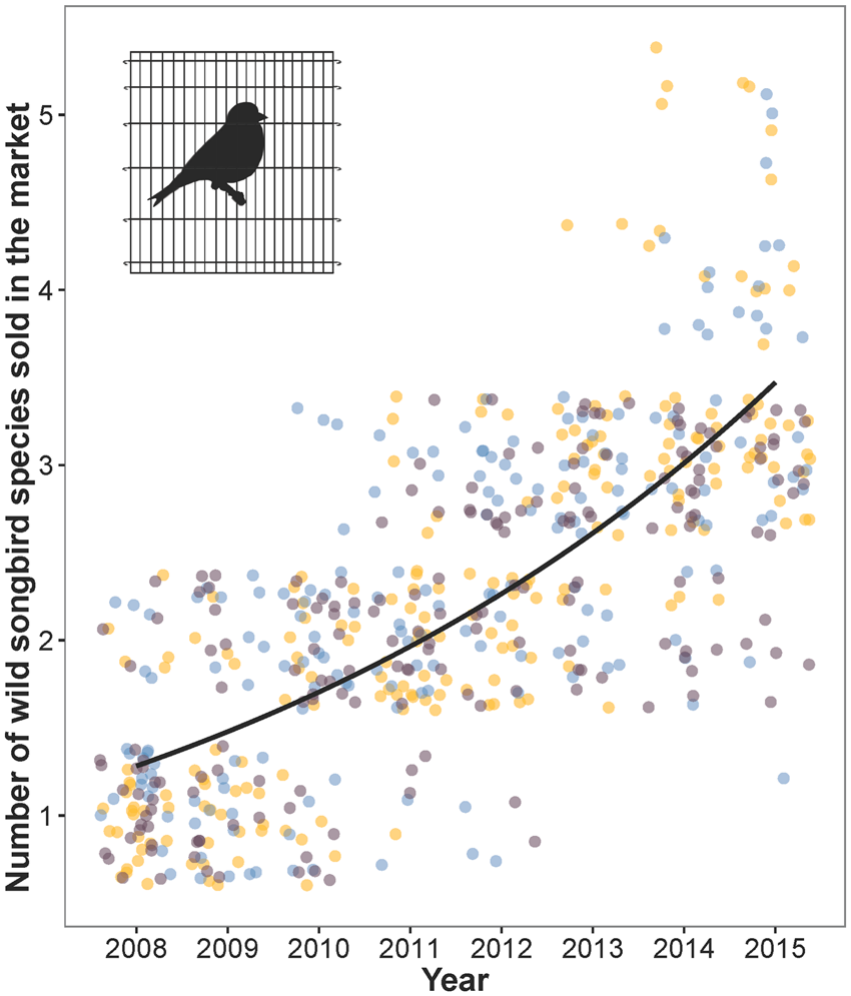
Changes in the number of wild species of songbirds regularly sold in the market between 2008 and 2016. The points were jittered to avoid overlap. Here, exotic species were not included. The fitted line is a Poisson regression (averaged across provinces). Colors refer to provinces; blue: Annaba, orange: Guelma and, brown: Skikda.

The use of mist nets as poaching tool in western Maghreb started in 2009–2010. Our survey conducted on 12 poachers from three Algerian provinces revealed that goldfinch trapping with mist nets during 2010–2012 captured 16 bird species besides European goldfinch, belonging to 10 families (Table 2). Of these species, five (31.25%) are migratory and the rest (68.75%) are resident. To make the connection between the use of mist nets and the increase of cage-bird species in the flea markets, we surveyed the number of cage-bird species sold in the flea markets of the same three Algerian provinces during 2008–2015. A checklist of species sold in the market in the same three Algerian provinces is shown in Supplementary Table 2. Interestingly, there was a significant increase of the number of cage-bird species during this period (GLM Poisson: slope = 0.142, z-value = 11.28, P < 0.0001) (Fig. 7). The average number of species a﻿cross provinces (﻿excluding exotic species) that are regularly sold in the flea markets was 1.18 ± 0.39 (±SD, range = 1–2, n = 75) in 2008 (before mist net poaching) and 3.27 ± 0.82 (±SD, range = 1–5, n = 69) in 2015 (after mist net poaching). Overall, five Palearctic Fringilid cage-birds were regularly recorded, of which four are resident and one is migratory. Importantly, they are all passerines that are caught as by-catch of the European goldfinch poaching, suggesting that the severe decline of the European goldfinch might have yielded new target species for domestication which perpetuates the poaching issues in the region.

**Table 2:** Checklist with trading and migratory status of species captured as by-catch during the European goldfinch trapping by mist nets in three Algerian provinces.

Common name	Scientific name	Family	Traded	Status
Eurasian collared dove	*Streptopelia decaocto*	Columbidae	No	Resident
Common cuckoo	*Cuculus canorus*	Cuculidae	No	Migratory
Eurasian siskin	*Spinus spinus*	Fringillidae	Yes	Migratory
European serin	*Serinus serinus*	Fringillidae	Yes	Resident
European greenfinch	*Chloris chloris*	Fringillidae	Yes	Resident
Tree pipit	*Anthus trivialis*	Motacillidae	No	Migratory
Black redstart	*Phoenicurus ochruros*	Muscicapidae	No	Migratory
European robin	*Erithacus rubecula*	Muscicapidae	No	Resident
Great tit	*Parus major*	Paridae	No	Resident
African blue tit	*Cyanistes teneriffae*	Paridae	No	Resident
House sparrow	*Passer domesticus*	Passeridae	No	Resident
Willow warbler	*Phylloscopus trochilus*	Phylloscopidae	No	Migratory
Sardinian warbler	*Sylvia melanocephala*	Sylviidae	No	Resident
Red crossbill	*Loxia curvirostra*	Fringillidae	Yes	Resident
Common chaffinch	*Fringilla coelebs*	Fringillidae	Yes	Resident
Common blackbird	Turdus merula	Turdidae	No	Resident

Algerian provinces are Guelma, Annaba, and Skikda, and are all located in the Northeast.

## Discussion

Our study shows that the domestication of the European goldfinch has had severe consequences on the dynamics of wild populations in western Maghreb and may threaten many other local and Afro-Palearctic migratory songbirds. We first found that the distribution range of the species has undergone rapid retraction during the past 26 years in western Maghreb. We estimated the total number of domesticated goldfinches of 6.3 million in Algeria, and about 15.6 million in the entire western Maghreb. We showed evidence that the currently existing populations of goldfinch are under huge poaching pressure by an established international trading system, which threatens the persistence of the species in the area. Finally, poaching of goldfinches is currently conducted within one of the most important flyways in the world using mainly mist nets which might affect several migratory bird populations flying through this region.

We estimated a loss of range distribution for the European goldfinch of nearly 57% across western Maghreb during the past 26 years. The species is probably extinct or very scarce in the wild in the eastern part of the study area, that is, in Tunisia and Algeria (except for the western part). In our knowledge, such rapid range fragmentation is rare in Palearctic passerines^31^. According to BirdLife International^17^, the population trends of the species since 1980 have been stable based on provisional data for 21 European countries. Knowing that goldfinch poaching is either absent or low in these countries compared to western Maghreb, our results reveal that intensive poaching could affect severely population dynamics and may ultimately lead to rapid extinction of an abundant species. Although poaching has probably played a major role in the extinction of many wild populations, other factors like climate change leading to severe drought and forest fires shrinking the potential habitats for the species might have exacerbated the rapid range retraction.

There are a few mutually non-exclusive hypotheses that may explain why Moroccan populations did not decline as much as Algerian and Tunisian populations. First, there might be differences in poaching pressure between the three countries of western Maghreb so that Moroccan natural populations of goldfinch were less exploited than Algerian and Tunisian populations during the 1990s and early 2000s. Second, the higher availability of forests (an important habitat for the species) in Morocco^32^ could have conferred a larger carrying capacity for the species and more resilience to population extinction^33^. Third, the connection with the European populations of goldfinch may also have contributed to the persistence of Moroccan populations. In fact, in terms of distance, Moroccan populations are closer to European populations with a minimum distance between the Spanish and Moroccan coast of 14 km at the Strait of Gibraltar. Thus, it is possible that regular colonization of Moroccan habitats by Spanish populations compensates, to some extent, the effects of intensive poaching^34^.

Our results showed that the price of the European goldfinch in the market, even corrected for the monthly per capita income, increased exponentially during the past 26 years. As it is the case in many species with economic value^5^, the price increased continually as the species became rarer. The expensiveness of the species was probably one of the factors that increased the poaching motivation and caused extinction of wild populations. In addition, it also places the remaining populations under high harvest risk since one goldfinch is currently worth nearly the third of the average monthly income. Hence, because of the low effectiveness of the conservation plans, many non-poachers probably became increasingly interested in capturing or trading the goldfinch given the current economic crisis and high unemployment rate that countries of the western Maghreb encounter. Further conservation policies should include an economic perspective in the elaboration of new laws by understanding the relationship between the demand (customer) and the supply (poacher)^35^. We suspect a combination of the snob effect^36^ ‒ people pay more money for rarer species^37–40^ and the bandwagon effect^36^ ‒ people’s valuation of the species increased its popularity among society. In other words, the increased economic value of the goldfinch following the decline of the wild populations intensified the poaching pressure, which in turn heightened the popularity and social value of the species, leading to the increase in the demand. Therefore, conservationists and policy makers should elaborate effective supply-side and demand-side antipoaching policies and carefully think about the potential interplay between them^35^.

We estimated an average of 6.3 million goldfinches domesticated for Algeria and 15.6 million for western Maghreb. This most likely represents the highest density of domesticated European goldfinches in the world. According to our estimates, nearly every family in the region possesses one caged individual, which reflects the cultural importance of the species to local people. Although our estimates were based on extrapolations from 15 provinces to the entire Algerian territory (48 provinces), we, nevertheless, carefully chose provinces that are well-distributed across the northern part of the country (east, center and west) where most Algerian population lives. Our extrapolation to western Maghreb (Tunisia, Algeria and Morocco) which was motivated by the similarity of the cultural importance and popularity of the goldfinch between the three countries needs further confirmation to overcome potential under- or overestimation biases. Surprisingly, a very low proportion of domesticated goldfinches are bred in captivity, which means that the primary supply for the pet owners are individuals that come from the flea markets, that is, those poached from wild populations. This lack of captive breeding combined with the large distribution of domestication for a species that has shown a severe range retraction is deeply alarming and requires enforcement of existing laws and raising awareness of the ecological consequences of poaching in general and the unsustainable practice of the cultural domestication of the bird in particular. Nonetheless, the cultural component of the domestication of the goldfinch has to be taken into account to solve the problem by applying actions that both satisfy people's needs and protect natural populations^41, 42^.

Our survey revealed the international illegal trading network of European goldfinch that occurs in western Maghreb. Due to the extinction of most Tunisian and Algerian natural populations of the species, representing the primary supply for the local cultural domestication, the demand for the species increased. Thus, poachers started to illegally export the species from Morocco, where populations are still flourishing, to Algeria and Tunisia. This places the last populations of the Maghreb under high extinction risks because the Moroccan populations of European goldfinch are harvested to meet the demands of three countries which triples the poaching pressure. Moreover, cases of exportation of the goldfinch from western Maghreb to Europe (France and Belgium) were recently reported, which enlarges the international trading system of the species^43^. This international network of European goldfinch trafficking has to be regulated rapidly by reinforcing surveillance at borders, before the species goes extinct in its extreme southern margin of the Palearctic.

The introduction of mist nets as a capture tool is the most alarming poaching aspect that we recorded in our study. In fact, as the species became rare, the effectiveness of ground nets and lime sticks reduced because individuals have to land to be trapped, which is not the case for mist nets. The efficiency, larger capture capacity, and invasive aspect of mist nets could induce substantial collateral damage to local and migratory species^26^. North Africa is an important avian region where several species of European birds fly through or stop over twice a year during the migration from breeding areas in Europe to wintering grounds in Africa. We recorded 16 passerine species captured as by-catch during the goldfinch poaching from only 12 poachers who used mist net trapping in Northeast Algeria. Therefore, the number of species that have been intercepted in the entire western Maghreb must be substantially higher, especially in Morocco where millions of birds cross Gibraltar to reach African wintering areas^26^. Although our data are only qualitative, they should sound the alarm for immediate actions. The fate of birds captured as by-catch remains unclear, but we have evidence that the number of traded songbirds in the market, including migratory species, increased during the last five years, which coincides with the introduction of mist nets in the wild. Therefore, it is likely that the rarity and expensiveness of the European goldfinch led local people to seek for alternative pet birds which in turn became the target of poaching. This may be the start of another round of extensive poaching not for one species, but for a large spectrum of wild songbirds, which puts the Palearctic migratory birds under unprecedented poaching threat in the region.

Although the species was listed in the nationally protected birds in the three countries of western Maghreb, the local conservation actions and politics of the species are still inefficient. The concentration of the conservation authorities is directed towards natural populations, international borders and national checkpoints. These measures are crucial, but do not treat the problem from its roots since the main trade center of European goldfinch is the weekly flea markets that are widespread in the region, representing the primary link between poachers, traders and customers. There, freshly captured wild birds are illegally sold all year long without any surveillance. Thus, future conservation strategies must firstly consider banning illegal trading of wild individuals in flea markets. In addition, other key urgent actions should include restoration planning, artificial breeding of the species, monitoring of wild populations, and sensitization of local people. First, the restoration of wild populations of European goldfinch in western Maghreb should be successful given the advantageous demographic proprieties of the species. In fact, the high reproductive rate of the species^44^ and the fast maturity of juveniles offer the species biological attributes that increase the recruitment probability, contribute highly to population growth^45–47^, and potentially result in recolonization of habitats and range expansion. Second, promoting artificial breeding of the species in captivity is a top priority because it offers a sustainable harvest solution that should produce enough individuals to meet the cultural and socio-economic needs for local people. Third, regular individual marking and monitoring of wild populations are crucial to assess the success of restoration plans, estimate demographic parameters (survival and fertility), assess dispersal of individuals, and determine population trends. Finally, a social sensitization scheme should be initiated to raise the awareness of people about the consequences of poaching on ecosystem functioning and human well-being.

## Methods

### Range dynamics

The study site is the western Maghreb which includes Tunisia, Algeria and Morocco. Because we were interested in the changes of the range distribution of European goldfinch during the past 26 years, we compiled data of the species occurrence in western Maghreb within 237 grids (25 × 25 km²) from poachers and field visits, and we built a detection/non-detection dataset which included observations before (1990–1991) and after the intensive poaching (2015–2016) in each site (grid) (Supplementary Fig. 2). The selection of the grids was based on our social network that we could establish for this project. The European goldfinch is easy to identify in the wild because of its unique and distinguishable morphological features with respect to other local passerines. Data collected for the period 1990–1991 were based on poachers recalling having observed goldfinches that they personally poached from the wild. We are confident that data obtained from people are accurate since we used the following criteria to increase the quality of detection data: (1) we selected those who used to own the bird and poach it in the wild (expert poachers), and thus the probability of false positive detection is very low; (2) observations/trapping were made when weather conditions were good, so there is no detection-bias related to weather; (3) we included only observers and poachers who made at least 10 visits/year/grid and stayed at least at least 2 hours in the field; (4) poachers were asked whether they observed the species and not whether they trapped it because in many cases the bird is observed but not trapped. To control for consistency in sampling efforts for the two study periods (1990–1991 and 2015–2016), we maintained a balanced sampling effort for both periods by limiting the number of observers (or poachers) to one observer/year/grid.

Since our purpose is to reveal changes of species occupancy resulting from 26 years of intensive poaching, we considered period (1990–1991 or 2015–2016) as a primary sampling occasion and year as a secondary sampling occasion. We used dynamic occupancy models using the R package unmarked^48^ in R 3.2.1^49^ to estimate the initial occupancy, extinction, colonization, and detection probabilities. We tested for potential relationships of these parameters with covariates namely vegetation density, elevation and longitude estimated with raster map analysis and interpolated digital elevation model using Global Mapper (version 15.0, http://www.bluemarblegeo.com/products/global-mapper.php) and MapInfo Professional software (version 15.0, http://www.pitneybowes.com/us/location-intelligence/geographic-information-systems/mapinfo-pro.html).

We conducted model selection using Akaike's information criterion (AIC) on models with increasing complexity in terms of covariates. Both linear and quadratic effects for covariates were fitted. We first fitted the model with constant parameters and compared it with a model where detection was time-dependent. After selecting which model was better, we added to detection probability (*p*) the linear and quadratic effects of vegetation cover and elevation. Then we repeated the operation for initial occupancy, extinction and colonization probabilities by increasing covariate complexity and performing AIC-based model selection^29^. The goodness of fit of the overall best model was assessed with a parametric bootstrapping approach^48^ based on a chi-square test statistic for the AIC-best model (P-value = 0.20) which indicated that there was no lack of fit and thus the model can be used for predictions. To obtain predictions (with statistical uncertainty) for initial occupancy, extinction and colonization and detection probabilities, we used the *predict* function in *unmarked*. Parametric bootstrap (replicates = 1000) was used to calculate confidence intervals for the initial occupancy, extinction and colonization probabilities using the *parboot* function in *unmarked*. We produced maps of predicted occupancy before (1990–1991) and after (2015–2016) the industrial poaching of the species with MapInfo Professional software (version 15.0, http://www.pitneybowes.com/us/location-intelligence/geographic-information-systems/mapinfo-pro.html) based on our best model.

### Temporal pattern of prices

To investigate the changes in the economic value of the European goldfinch in the region, long-term traders in eight flea markets from different Algerian provinces (Oran, Algiers, Blida, Annaba, Guelma, Jijel, Skikda, and Souk Ahras) were asked for the yearly mean price of adults from 1990 to 2015. As the economy and purchasing power changed considerably in Algeria during the past two decades, we also calculated the price corrected for the per capita monthly income (PCI) by dividing the price of the goldfinch of a given year by the monthly per capita income of the same year^50^. The currency was converted to US dollars^51^. The temporal pattern of the price and the PCI were fitted with exponential regression models.

### Domestic population estimation

To estimate the number of European goldfinches currently in captivity in Algeria, we selected 15 provinces well-distributed across the Northern part of the country (five in the east, five in the center and five in the west, Supplementary Fig. 3; Supplementary Table 3). In each province, we selected typically 3–5 districts where 3–7 buildings were sampled (Supplementary Table 4). Each building has a number of apartments and we assumed that each apartment has one family which is the case in Algeria and western Maghreb. For instance, a typical building has 10 to 12 apartments which corresponds to 10 to 12 families. Overall, 83 (982 families), 52 (949 families) and 51 (790 families) buildings were sampled from the East, center and West, respectively. Given our sample size and geographic coverage, we assume that our sampling do not lead to biased estimations of the average number of goldfinches owned per family and the number of owners that carry out captive breeding. We calculated the total number of goldfinches domesticated in Algeria by multiplying the average number of goldfinches owned per family by the estimated number of families in the country (Supplementary Table 5). Because the cultural importance and popularity of the European goldfinch is similar in the three countries of western Maghreb, we also estimated the total number of individuals held in captivity for the entire region using Algerian estimates.

### International trading

Given that all or most Tunisian and Algerian populations became extinct or very rare during the last decade and that Moroccan populations are still flourishing, poachers have illegally transported the goldfinch from Morocco to Algeria and Tunisia for trading during the last decade. To reveal the amplitude of the trading network between the three countries, we collected data of the number of seizures, the destination of birds, and the number of goldfinches that the conservation authority seized between January 2008 and August 2016 in Algeria from the DGF (Direction générale des forêts) and gendarmerie. The number of goldfinches and occasions of interceptions were mapped with MapInfo Professional software (version 15.0, http://www.pitneybowes.com/us/location-intelligence/geographic-information-systems/mapinfo-pro.html). We calculated the total number of individuals seized and the average number of individuals per seizure during the nine years (2008–2016).

### Bycatch

To obtain a list of species captured as by-catch with mist nets, we conducted a survey on 12 poachers from three Algerian provinces (Guelma, Skikda, and Annaba). We obtained data only for the period 2010–2012. We were not able to gather quantitative data regarding the number of individuals captured for each species since poachers were careful﻿ and not willing to fully cooperate on an illegal act punishable by law. To investigate the potential relationship between the changes in the number of cage-bird species sold in the market and mist net trapping, each week we recorded cage-bird species that are sold in the weekly flea markets of the same three provinces at least 20 times every year from 2008 to 2015 (Supplementary Table 6). We excluded all exotic species and included only species that are locally resident or migratory. Then, we estimated the temporal pattern of the number of cage-bird species that were sold in the market during 2008–2015 with a generalized Poisson model using year as a fixed effect.

## Electronic supplementary material

Supplementary Material
