# Supplementary material for: Unravelling the drastic range retraction of an emblematic songbird of North Africa: potential threats to Afro-Palearctic migratory birds
Source: Sci Rep. 2017 Apr 24;7:1092. doi: 10.1038/s41598-017-01103-w (PMC5430695; doi:10.1038/s41598-017-01103-w)
Supplement: Supplementary file 1 — Supplementary Material [file 41598_2017_1103_MOESM1_ESM.doc]

**Supplementary information**

**Unravelling the drastic range retraction of an emblematic songbird of North Africa: potential threats to Afro-Palearctic migratory birds**

Rassim Khelifa^1*^, Rabah Zebsa^2^, Hichem Amari^3^, Mohammed Khalil Mellal^4^, Soufyane Bensouilah^3^, Abdeldjalil Laouar^5^, Hayat Mahdjoub^1^

Corresponding author: Rassim Khelifa

e-mail: [rassimkhelifa@gmail.com](mailto:rassimkhelifa@gmail.com)

Phone number: 0041786581986

**Supplementary Figures**


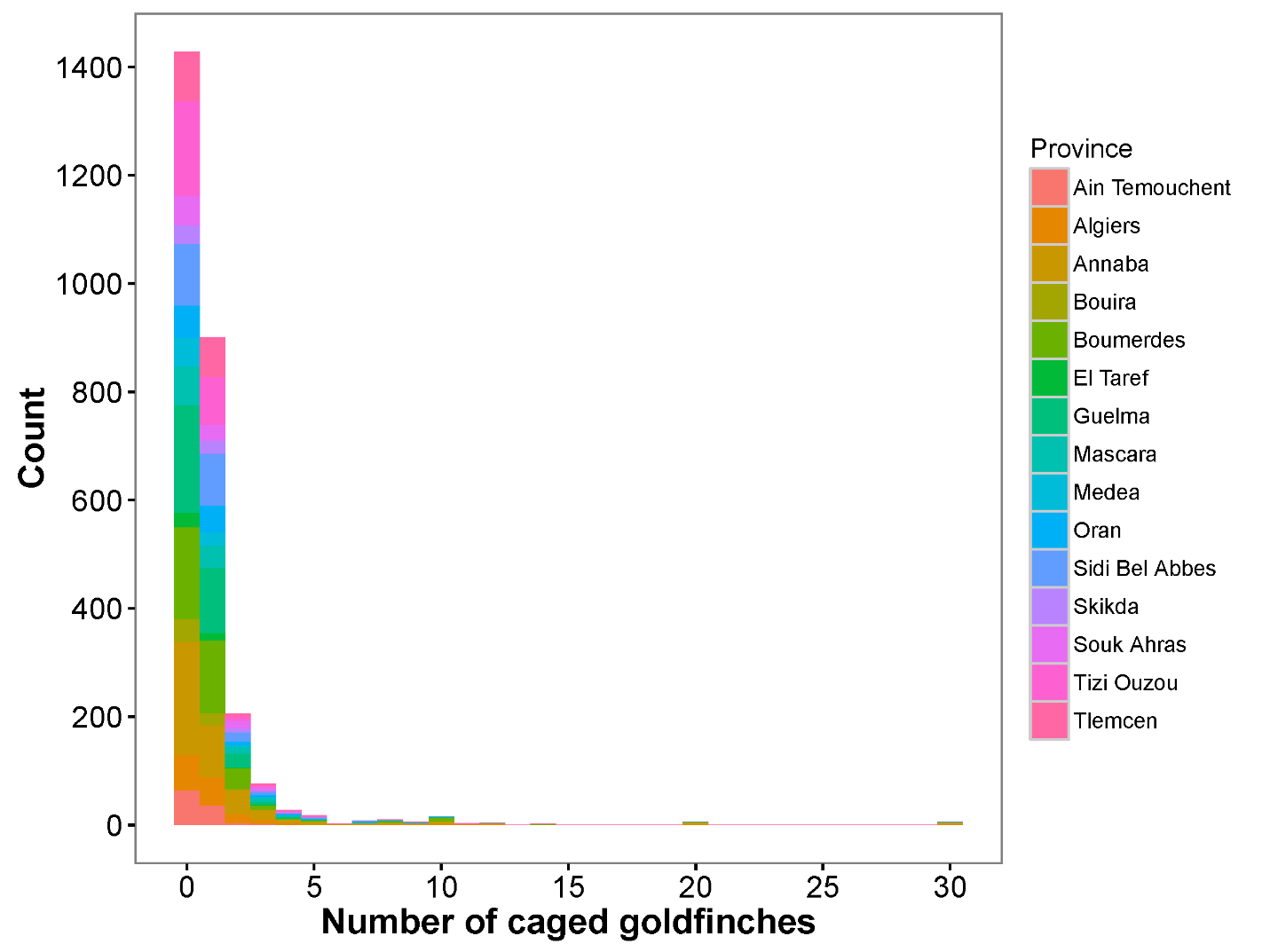


**Supplementary Figure 1.** Frequency distribution of the number of caged birds per family in 15 Algerian provinces. The total sample size was 2721 families and the average number of caged goldfinches was 0.925 (range: 0-30 goldfinches).


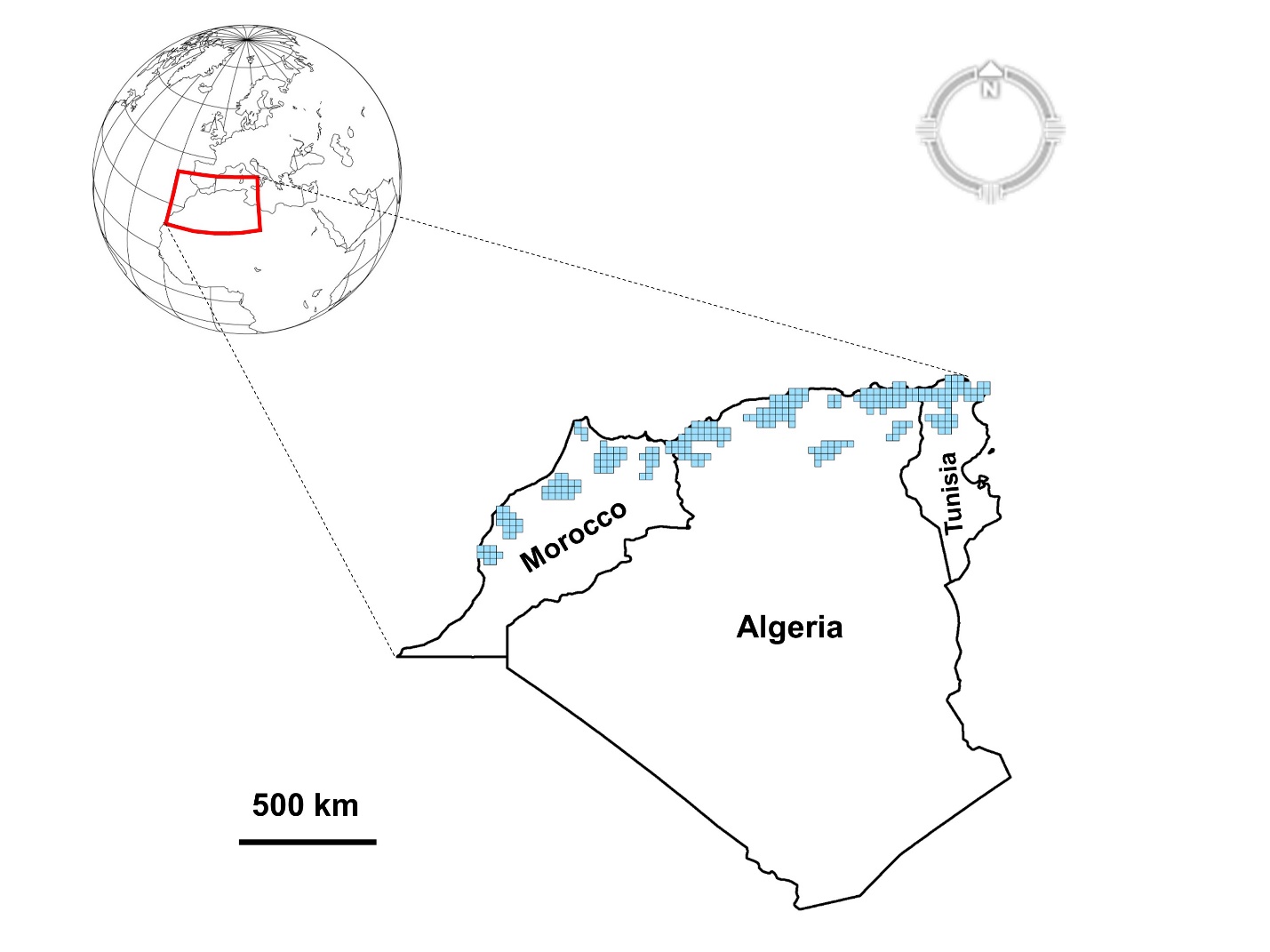


**Supplementary Figure 2.** Study area with sampling grids for estimations of the changes of distribution of the European goldfinch (*Carduelis carduelis*) after 26 years of intensive poaching. Each blue grid is 25×25 km² where detection/non-detection data of the species were obtained for 1990, 1991, 2015 and 2016. Note that the grids are mostly distributed across the littoral part Morocco, Algeria and Tunisia because our sampling was based on prior knowledge of species habitat preferences. In fact, since the species prefers vegetated areas and avoids arid lands, the southern parts of the countries were omitted in our analyses. The map was created with MapInfo Professional software (version 15.0, http://www.pitneybowes.com/us/location-intelligence/geographic-information-systems/mapinfo-pro.html).


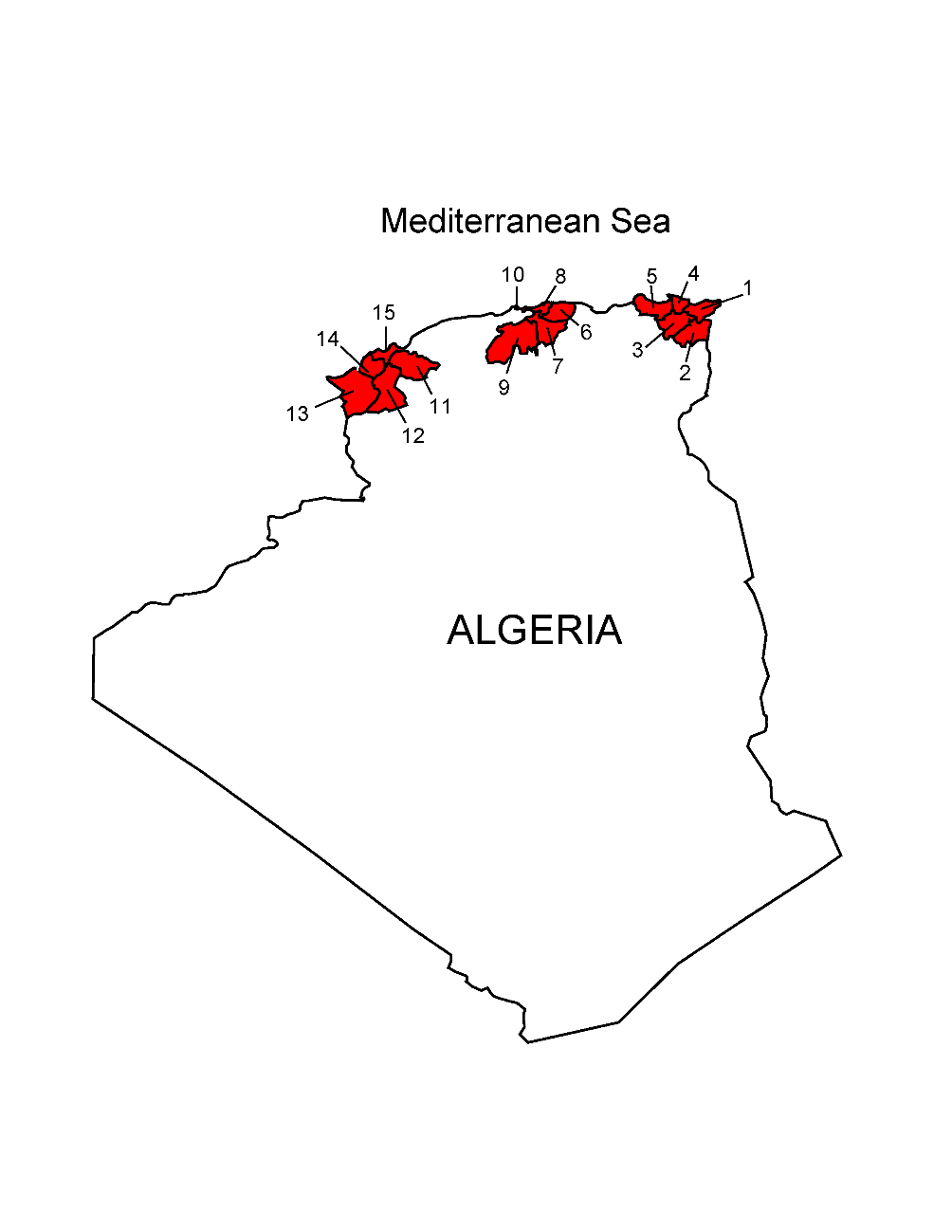


**Supplementary Figure 3.** Study provinces in Algeria for the estimation of the number of captive goldfinch. We refer to province 1 to 5 as east Algeria, 6 to 10 as central Algeria, and 11 to 15 as west Algeria (Supplementary table 3). We sampled only the northern part of the country where the Algerian population is concentrated. The map was created with MapInfo Professional software (version 15.0, http://www.pitneybowes.com/us/location-intelligence/geographic-information-systems/mapinfo-pro.html).

**Supplementary Tables**

**Supplementary Table 1.** Results of model selection for the European goldfinch (*Carduelis carduelis*) range dynamics from 1990-1991 to 2015-2016 in western Maghreb. The sequential approach of model selection here is structured such that it first compares a fully time-dependent and a fully constant model, then it compares candidate models for detection (p), initial occupancy (Ψ1), extinction (**ε**), and colonization (**ϒ**). The best model is selected based on the Akaike’s information criterion (AIC) and parsimony. The model retained is shown in bold. nPars refers to the number of estimated parameters. Covariates are abbreviated as E (elevation), Veg (vegetation cover), Long (longitude), and Y (year). Quadratic terms are denoted by a superscript 2 and interactions by a colon. Dot refers to a constant model.

| **Model** | **nPars** | **AIC** | **ΔAIC** | **AICwt** |
| --- | --- | --- | --- | --- |
| **(a) Modelling time dependence in parameters** |  |  |  |  |
| **Ψ1(.) ϒ(.) ε(.) p(Y)** | **5** | **531.54** | **0.00** | **0.99954** |
| Ψ1(.) ϒ(.) ε(.) p(.) | 4 | 546.91 | 15.36 | 0.00046 |
|  |  |  |  |  |
| **(b) Modelling patterns in detection probability (p)** |  |  |  |  |
| **Ψ1(.) ϒ(.) ε(.) p(Y + Veg + Veg² + E + E² + Veg : E + Veg : E² )** | **11** | **301.46** | **0.00** | **1.0e+00** |
| Ψ1(.) ϒ(.) ε(.) p(Y + Veg + E + E² + Veg : E + E:Veg² ) | 10 | 330.61 | 29.15 | 4.7e-07 |
| Ψ1(.) ϒ(.) ε(.) p(Y + Veg + E + E² + Veg : E ) | 9 | 336.66 | 35.21 | 2.3e-08 |
| Ψ1(.) ϒ(.) ε(.) p(Y + Veg + E + E² + Veg : E + Veg : E² ) | 10 | 338.13 | 36.67 | 1.1e-08 |
| Ψ1(.) ϒ(.) ε(.) p(Y + Veg + E + E²) | 8 | 349.58 | 48.12 | 3.6e-11 |
| Ψ1(.) ϒ(.) ε(.) p(Y + Veg + Veg²) | 7 | 378.03 | 76.57 | 2.4e-17 |
| Ψ1(.) ϒ(.) ε(.) p(Y + Veg + E ) | 7 | 395.60 | 94.15 | 3.6e-21 |
| Ψ1(.) ϒ(.) ε(.) p(Y + Veg ) | 6 | 428.47 | 127.02 | 2.6e-28 |
| Ψ1(.) ϒ(.) ε(.) p(Y + E + E²) | 7 | 529.17 | 227.71 | 3.6e-50 |
| Ψ1(.) ϒ(.) ε(.) p(Y + E ) | 6 | 531.03 | 229.58 | 1.4e-50 |
| Ψ1(.) ϒ(.) ε(.) p(Y) | 5 | 531.54 | 230.09 | 1.1e-50 |
|  |  |  |  |  |
| **(c) Modelling patterns in first-year occupancy probability (Ψ)** |  |  |  |  |
| **Ψ1( Veg + E + E²) ϒ(.) ε(.) p(Y + Veg + Veg² + E + E² + Veg : E + Veg : E² )** | **14** | **254.83** | **0.00** | **4.9e-01** |
| Ψ1( Veg + E + E² + E:Veg²) ϒ(.) ε(.) p(Y + Veg + Veg² + E + E² + Veg : E + Veg : E² ) | 15 | 254.97 | 0.13 | 4.6e-01 |
| Ψ1( Veg + E) ϒ(.) ε(.) p(Y + Veg + Veg² + E + E² + Veg : E + Veg : E² ) | 13 | 259.76 | 4.92 | 4.2e-02 |
| Ψ1( Veg) ϒ(.) ε(.) p(Y + Veg + Veg² + E + E² + Veg : E + Veg : E² ) | 12 | 286.43 | 31.60 | 6.8e-08 |
| Ψ1( E ) ϒ(.) ε(.) p(Y + Veg + Veg² + E + E² + Veg : E + Veg : E² ) | 12 | 302.21 | 47.37 | 2.6e-11 |
| Ψ1( 1 ) ϒ(.) ε(.) p(Y + Veg + Veg² + E + E² + Veg : E + Veg : E² ) | 8 | 349.58 | 94.74 | 1.3e-21 |
|  |  |  |  |  |
| **(d) Modelling patterns in extinction probability (ε)** |  |  |  |  |
| **Ψ1( Veg + E + E²) ϒ(.) ε(Long + Long² + E) p(Y + Veg + Veg² + E + E² + Veg : E + Veg : E² )** | **17** | **180.72** | **0.00** | **8.2e-01** |
| Ψ1( Veg + E + E²) ϒ(.) ε(Long + Long²) p(Y + Veg + Veg² + E + E² + Veg : E + Veg : E² ) | 16 | 183.84 | 3.12 | 1.7e-01 |
| Ψ1( Veg + E + E²) ϒ(.) ε(Long + Veg + E + E²) p(Y + Veg + Veg² + E + E² + Veg : E + Veg : E² ) | 18 | 191.20 | 10.47 | 4.3e-03 |
| Ψ1( Veg + E + E²) ϒ(.) ε(Long + Long² + E + E²) p(Y + Veg + Veg² + E + E² + Veg : E + Veg : E² ) | 18 | 191.21 | 10.48 | 4.3e-03 |
| Ψ1( Veg + E + E²) ϒ(.) ε(Long) p(Y + Veg + Veg² + E + E² + Veg : E + Veg : E² ) | 15 | 194.02 | 13.30 | 1.1e-03 |
| Ψ1( Veg + E + E²) ϒ(.) ε(Long + Veg) p(Y + Veg + Veg² + E + E² + Veg : E + Veg : E² ) | 16 | 195.30 | 14.57 | 5.6e-04 |
| Ψ1( Veg + E + E²) ϒ(.) ε(Long * Veg) p(Y + Veg + Veg² + E + E² + Veg : E + Veg : E² ) | 17 | 197.30 | 16.58 | 2.1e-04 |
| Ψ1( Veg + E + E²) ϒ(.) ε(.) p(Y + Veg + Veg² + E + E² + Veg : E + Veg : E² ) | 14 | 254.83 | 74.11 | 6.6e-17 |
|  |  |  |  |  |
| **(e) Modelling patterns in colonization probability (ϒ)** |  |  |  |  |
| **Ψ1( Veg + E + E²) ϒ(.) ε(Long + Long² + E) p(Y + Veg + Veg² + E + E² + Veg : E + Veg : E² )** | **17** | **180.72** | **0.00** | **0.516** |
| Ψ1( Veg + E + E²) ϒ(Veg) ε(Long + Long² + E) p(Y + Veg + Veg² + E + E² + Veg : E + Veg : E² ) | 18 | 182.72 | 2.00 | 0.190 |
| Ψ1( Veg + E + E²) ϒ(E) ε(Long + Long² + E) p(Y + Veg + Veg² + E + E² + Veg : E + Veg : E² ) | 18 | 182.72 | 2.00 | 0.190 |
| Ψ1( Veg + E + E²) ϒ(Veg + Veg²) ε(Long + Long² + E) p(Y + Veg + Veg² + E + E² + Veg : E + Veg : E² ) | 19 | 184.73 | 4.01 | 0.069 |
| Ψ1( Veg + E + E²) ϒ(E + E²) ε(Long + Long² + E) p(Y + Veg + Veg² + E + E² + Veg : E + Veg : E² ) | 19 | 186.78 | 6.05 | 0.025 |
| Ψ1( Veg + E + E²) ϒ(E + E² + Veg) ε(Long + Long² + E) p(Y + Veg + Veg² + E + E² + Veg : E + Veg : E² ) | 20 | 188.50 | 7.78 | 0.011 |

**Supplementary Table 2.** Checklist of species sold in the market in Algeria with trading and migratory status. Data were collected from three markets from three provinces. 1: Guelma, 2: Skikda, 3: Annaba.

| **Common name** | **Scientific name** | **Family** | **Status** | **Market** |
| --- | --- | --- | --- | --- |
| **European serin** | *Serinus serinus* | Fringillidae | Resident | 1, 2, 3 |
| **European greenfinch** | *Chloris chloris* | Fringillidae | Resident | 1, 2 |
| **Eurasian siskin** | *Spinus spinus* | Fringillidae | Migratory | 1, 3 |
| **European goldfinch** | *Carduelis carduelis* | Fringillidae | Resident | 1, 2, 3 |
| **Common chaffinch** | *Fringilla coelebs* | Fringillidae | Resident | 1, 3 |

**Supplementary Table 3.** Algerian provinces sampled for domestic population estimation of the European goldfinch. See Supplementary Fig. 2 for geographic location of provinces.

| **Region** | **Province** | **Province code** |
| --- | --- | --- |
| **East** | El Taref | 1 |
| **East** | Souk Ahras | 2 |
| **East** | Guelma | 3 |
| **East** | Annaba | 4 |
| **East** | Skikda | 5 |
| **Center** | Tizi Ouzou | 6 |
| **Center** | Bouira | 7 |
| **Center** | Boumerdes | 8 |
| **Center** | Medea | 9 |
| **Center** | Algiers | 10 |
| **West** | Mascara | 11 |
| **West** | Sidi Bel Abbes | 12 |
| **West** | Tlemcen | 13 |
| **West** | Ain Temouchent | 14 |
| **West** | Oran | 15 |

**Supplementary Table 4.** Number of families and buildings sampled in 15 Algerian provinces. These data were used to estimate the average number of European goldfinch kept in captivity and the average number of owners performing artificial breeding in Algeria.

| **Region** | **Wilaya** | **No.families** | **N.o.buildings** |
| --- | --- | --- | --- |
| **Center** | Algiers | 159 | 11 |
| **Center** | Bouira | 68 | 7 |
| **Center** | Boumerdes | 364 | 12 |
| **Center** | Medea | 82 | 10 |
| **Center** | Tizi Ouzou | 276 | 12 |
| **East** | Annaba | 400 | 29 |
| **East** | El Taref | 44 | 6 |
| **East** | Guelma | 360 | 26 |
| **East** | Skikda | 68 | 7 |
| **East** | Souk Ahras | 110 | 15 |
| **West** | Ain Temouchent | 106 | 8 |
| **West** | Mascara | 136 | 10 |
| **West** | Oran | 134 | 11 |
| **West** | Sidi Bel Abbes | 236 | 11 |
| **West** | Tlemcen | 178 | 11 |
|  | **Total** | 2721 | 186 |

**Supplementary Table 5.** Total population size and the number of families in Morocco, Algeria and Tunisia in 2016. Data on total population size of 2016 were obtained from The World Bank (The World Bank 2016).

| **Country** | **Population** | **N.o.families** |
| --- | --- | --- |
| **Morocco** | 33655786 | 7272221 |
| **Algeria** | 40263711 | 6870269 |
| **Tunisia** | 11134588 | 1927649 |

We estimated the number of families in 2016 for each country where data are not yet available based on the latest estimates (2014 for Morocco ^1^ and Tunisia ^2^ and 2011 for Algeria ^3^) using this formula:


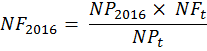


where NF is the number of families, NP is the total population size, t is year when data are last available.

**Supplementary Table 6. Number of visits and total number of wild cage-bird species regularly recorded in flea market of three Algerian provinces between 2008 and 2015. Since flea markets are weekly, the number of visits corresponds to the number of weeks sampled every year. The total number of cage-bird species excludes all non-Palearctic (exotic) species and includes only species that are locally resident or migratory.**

| **Year** | **Province** | **N.o.visits** | **Tot. Species** |
| --- | --- | --- | --- |
| **2008** | Annaba | 21 | 2 |
| **2008** | Guelma | 27 | 2 |
| **2008** | Skikda | 27 | 2 |
| **2009** | Annaba | 23 | 2 |
| **2009** | Guelma | 24 | 2 |
| **2009** | Skikda | 21 | 2 |
| **2010** | Annaba | 26 | 3 |
| **2010** | Guelma | 20 | 2 |
| **2010** | Skikda | 26 | 2 |
| **2011** | Annaba | 22 | 3 |
| **2011** | Guelma | 31 | 3 |
| **2011** | Skikda | 22 | 3 |
| **2012** | Annaba | 25 | 3 |
| **2012** | Guelma | 23 | 3 |
| **2012** | Skikda | 24 | 3 |
| **2013** | Annaba | 26 | 3 |
| **2013** | Guelma | 25 | 4 |
| **2013** | Skikda | 22 | 3 |
| **2014** | Annaba | 21 | 4 |
| **2014** | Guelma | 29 | 5 |
| **2014** | Skikda | 27 | 3 |
| **2015** | Annaba | 20 | 5 |
| **2015** | Guelma | 29 | 5 |
| **2015** | Skikda | 20 | 3 |

**Supplementary References**

1 Bulletin Officiel du Royaume du Maroc. Population légale d'après les résultats du RGPH 2014. **6354** (2015) (Date of access: 15/12/2016).

2 Institut National de la Statistique. Populations, logements et ménages par unités administratives et milieux. **Volume I** (2015) (Date of access: 15/12/2016).

3 Office Nationale des Statistiques. 5e Recensement Général de la Population et de l’Habitat, 2008: armature urbaine. **163** (2011) (Date of access: 15/12/2016).
